# Seasonal Variations in Slipped Capital Femoral Epiphysis

**DOI:** 10.3390/children13060772

**Published:** 2026-06-02

**Authors:** Carter James Kornak White, Xue-Cheng Liu, Scott E. Van Valin

**Affiliations:** 1 School of Medicine, Medical College of Wisconsin, Milwaukee, WI 53226, USA; cawhite@mcw.edu; 2 Department of Orthopedic Surgery, Children’s Wisconsin, Medical College of Wisconsin, Greenfield, WI 53227, USA; xcliu@mcw.edu

**Keywords:** SCFE, slipped capital femoral epiphysis, seasonality, Area Deprivation Index, socioeconomic deprivation, pediatric orthopaedics, slip severity, adolescent hip disorders, social determinants of health

## Abstract

Background: Slipped capital femoral epiphysis (SCFE) is a common adolescent hip disorder occurring during the pubertal growth spurt, and prior studies have demonstrated regional seasonal variation in presentation volume. The relationship between seasonality, socioeconomic deprivation, and disease severity remains incompletely characterized. This study investigated whether SCFE presentation volume demonstrates seasonal variation and whether seasonality is associated with socioeconomic deprivation, body mass index (BMI), and radiographic severity. Methods: We retrospectively reviewed children newly diagnosed with SCFE at a single institution in Wisconsin (USA) between January 2012 and March 2024. Variables included age, sex, race, Area Deprivation Index (ADI), BMI, month of symptom onset, Southwick slip angle, stability, chronicity, and symptom duration. Presentation volume was analyzed using 3-month rolling averages and sinusoidal regression with 12-month periodicity. Associations among ADI, BMI, and slip angle were assessed using linear regression and Spearman’s correlation. Results: In total, 122 of 160 patients met the inclusion criteria. SCFE presentation volume demonstrated significant seasonality (*p* < 0.05). Lower-volume months were associated with higher neighborhood deprivation and greater radiographic severity (*p* < 0.05). ADI correlated with slip angle (r = 0.29, *p* < 0.05) and BMI (r = 0.33, *p* < 0.05), whereas BMI was not significantly associated with slip angle. Chronic slips demonstrated greater slip angles than acute presentations (*p* < 0.05). Conclusions: SCFE presentation volume in Wisconsin follows a significant seasonal pattern, and social determinants of health may influence the timing of presentation and disease severity.

## 1. Introduction

Slipped capital femoral epiphysis (SCFE) is a common pediatric hip disorder that can lead to femoroacetabular impingement and early osteoarthritis if diagnosis and stabilization are delayed [1,2,3,4]. Typical symptoms include hip, thigh, or knee pain and a limp, and radiographic evaluation uses anteroposterior and frog-leg lateral pelvis views. Slip severity is commonly quantified with the Southwick slip angle [3,4].

Seasonal variation in SCFE presentation has been described across multiple healthcare systems, although reported timing of peak presentation varies by region and latitude [5,6,7,8,9,10,11]. Proposed mechanisms include seasonal shifts in vitamin D and growth velocity [4,9,12,13]. Clarifying whether seasonal variation exists within a given region may help clinicians maintain appropriate diagnostic suspicion during higher-risk periods and contextualize disease severity at presentation. Social determinants of health may also influence SCFE risk and outcomes; however, limited data on delayed care or access are present [14]. The Area Deprivation Index (ADI) is a neighborhood-level composite measure of socioeconomic disadvantage derived from census indicators of income, education, employment, and housing. Higher deprivation may relate to delayed access to evaluation and imaging, with downstream effects on slip severity at presentation [15].

The objectives of this study were to evaluate whether SCFE presentation in a single-center Wisconsin cohort demonstrates seasonal variation and whether neighborhood socioeconomic deprivation and radiographic severity (Southwick slip angle) vary by season of presentation. Secondary objectives were to examine simple associations between socioeconomic deprivation, body mass index, and radiographic severity at presentation.

## 2. Materials and Methods

### 2.1. Study Design and Setting

This retrospective cohort study included children and adolescents newly diagnosed with SCFE in Wisconsin (USA) at a single institution between January 2012 and March 2024. The institutional review board approved this study with a waiver for informed consent due to minimal risk and its retrospective design.

### 2.2. Participants

Patients were identified through single-center institutional clinical records using diagnostic codes and radiographic confirmation. Inclusion criteria included a diagnosis of SCFE during the study period and availability of key variables, including month of symptom onset and radiographic assessment. Exclusion criteria included traumatic or atypical slips (e.g., associated with endocrinopathy or renal osteodystrophy) and insufficient data for primary analyses.

### 2.3. Variables and Definitions

Demographic variables included age, sex, and race. Clinical variables included BMI at presentation, stability, chronicity, and symptom duration. Stability was defined by the ability to bear weight at presentation [2,4]. Chronicity was classified clinically, and symptom duration was recorded in days from reported onset to diagnosis. Radiographic severity was quantified by the Southwick slip angle measured on frog-leg lateral radiographs [3,4]. Neighborhood socioeconomic disadvantage was measured using the Area Deprivation Index (ADI). Patient home geographic information was used to assign ADI values from the publicly available Neighborhood Atlas data set [16]. This was chosen over other economic proxies such as the social vulnerability index (SVI) as the ADI represents more wholistic, long-term socioeconomic factors. Additionally, insurance type and Medicaid data were inconsistently reported in our data set, making it unfeasible to report on. Missing data were handled using available-case analysis. Patients with missing values for a specific variable were excluded only from analyses involving that variable. The number of available observations is reported in the corresponding tables where applicable.

### 2.4. Outcomes

Primary outcomes were (1) monthly SCFE presentation volume by month of symptom onset and (2) seasonal variation in mean ADI and mean Southwick slip angle at presentation. Secondary outcomes included associations among ADI, BMI, and slip angle and associations involving symptom duration and maturity measures.

### 2.5. Statistical Analysis

Cosinor analysis was used to evaluate seasonal variation in SCFE incidence over a 12-month period, which is a commonly used method for detecting and characterizing rhythmic or seasonal patterns in longitudinal and time-series data. In cosinor analysis, monthly case counts were modeled using a sinusoidal regression function with a fixed 12-month periodicity to assess cyclic temporal patterns. The model estimated the mesor (rhythm-adjusted mean), amplitude (magnitude of seasonal variation), and acrophase (timing of the peak occurrence). Statistical significance of seasonality was assessed using the fitted cosine and sine terms within the regression model. This approach is appropriate for testing seasonal variation in SCFE presentation because prior studies have demonstrated annual seasonal patterns in SCFE incidence. These are graphically represented with the x-axis month representing the centered 3-month average (previous month, the labeled month, and the following month). Continuous variables were evaluated using linear regression and Spearman’s correlation; nonparametric analyses were used when distributional assumptions were not met. The median (IQR) is reported for appropriate data characteristics. The Kruskal–Wallis rank sum test, Fisher’s exact test, and Pearson’s chi-squared test were used to determine significance. Statistical significance was defined as *p* < 0.05.

### 2.6. Writing Assistance

The editing tool (Grammarly, Version 14.1270.0) was used in the process of writing this manuscript for grammar, spelling, and clarity suggestions. It was not used for content or citation generation [17].

## 3. Results

### 3.1. Cohort Characteristics

Of 160 patients screened, 122 met the inclusion criteria. The analytic cohort included 55 female and 67 male patients with a mean age of 11.99 years. The mean follow-up time was 22.4 months. Demographic and clinical characteristics are summarized in Table 1. A significant difference was observed only in duration of symptoms throughout the seasons (*p* < 0.05). All other variables did not reach significance.

### 3.2. Seasonal Variation in SCFE Presentation

**Figure 1:**
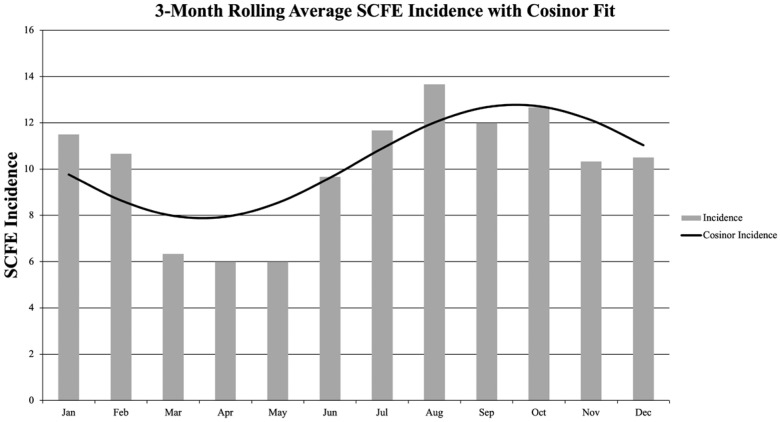
Three-month rolling average presentation volume by month with cosinor regression fit.

Sinusoidal regression demonstrated statistically significant seasonality in SCFE presentation volume (*p* < 0.05). Presentation volume was higher from July through January compared with February through June (*p* < 0.05) (see Figure 1).

### 3.3. Seasonal Variation in ADI and Slip Severity

**Figure 2:**
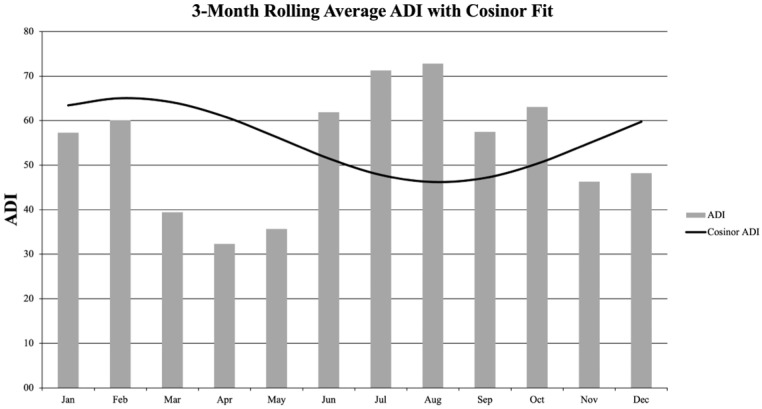
Three-month rolling average Area Deprivation Index (ADI) of patients at presentation by month with cosinor regression fit.

**Figure 3:**
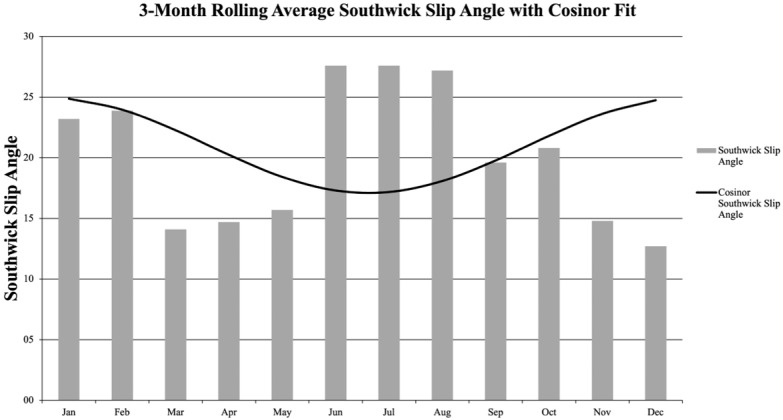
Three-month rolling average Southwick slip angle of patients at presentation by month with cosinor regression fit.

Sinusoidal regression demonstrated statistically significant seasonality in mean ADI at presentation and mean Southwick slip angle (both *p* < 0.05) (See Figure 2). Lower-presentation volume months (February through June) were characterized by higher mean ADI values and greater mean slip angles than higher-presentation volume months (July through January) (see Figure 3).

### 3.4. Associations Among ADI, BMI, and Slip Angle

ADI demonstrated modest positive associations with both BMI and slip severity. ADI correlated with slip angle (r = 0.28, *p* = 0.013) and with BMI (r = 0.315, *p* < 0.002). BMI was not significantly associated with slip angle (*p* > 0.05). Regression and correlation results are summarized in Table 2.

### 3.5. Chronicity, Symptom Duration, and Severity

The median duration of symptoms in days for Closed Triradiate Cartilage was 75 compared to 28 for Open Triradiate Cartilage (*p* = 0.004). Acute and chronic slips demonstrated similar median Southwick slip angles (20° and 21°, respectively), whereas acute-on-chronic slips exhibited a significantly higher median angle (34°) (*p* = 0.015).

## 4. Discussion

In this Wisconsin cohort, SCFE presentation volume demonstrated significant seasonality, with higher presentation volume from midsummer through midwinter. Seasonal variation has been observed in prior reports, although the timing of peak presentation has differed across regions and study designs [5,6,7,8,9,10,11]. Two meta-analyses performed on populations > 40° N reported a peak in presentation volume in August [9,18]. Our data show a peak in September, slightly different from that in previously reported data. However, the data set used in this study was limited to Wisconsin and smaller. Importantly, our findings extend prior work by demonstrating that seasonality was not limited to presentation volume alone.

In addition to variation in presentation volume, case characteristics also varied seasonally with higher slip angles and ADI scores seen in the winter months. Mean ADI and mean slip angle followed significant seasonal patterns, with greater neighborhood deprivation and radiographic slip severity being observed during lower-presentation volume months. Although overall presentation volume was lower in some months, those months were characterized by a higher mean ADI and greater radiographic severity, suggesting that seasonal patterns in presentation volume do not fully capture clinically important differences in case mix across the year. This inverse relationship between presentation volume and severity suggests that seasonal trends may mask clinically important differences in the populations presenting at different times of year. These findings suggest that periods of lower overall presentation volume may still warrant vigilance for more severe presentations in those with socioeconomic vulnerability. Several mechanisms may contribute to seasonality, including seasonal changes in activity levels, vitamin D status, and growth velocity. Each of these factors may interact with underlying mechanical vulnerability of the proximal femoral physis.

ADI was modestly associated with both BMI and slip severity. Although these associations explained a relatively small proportion of variance, their magnitude is consistent with expectations for multifactorial orthopedic conditions influenced by both biologic and social factors. The association between deprivation and BMI is consistent with prior epidemiological data [19]. The relation between obesity and SCFE risk has been well demonstrated [4,20]. The association between deprivation and slip severity may reflect barriers to timely evaluation, imaging, and referral, with delayed diagnosis allowing progressive deformity [15]. These barriers may include limited access to primary care, delayed subspecialty referral, or competing socioeconomic priorities that defer medical evaluation. Notably, BMI did not predict slip severity, which is consistent with prior work suggesting that BMI does not reliably correlate with slip severity once SCFE occurs [21].

Chronic slips demonstrated higher slip angles than acute presentations, reinforcing the importance of early recognition and prompt imaging. This finding supports the concept that slip severity reflects cumulative physeal displacement over time rather than an inherently more aggressive disease phenotype. Diagnostic delay remains a persistent issue in SCFE, particularly when symptoms are atypical or referred to the knee [3,4]. Maintaining a high index of suspicion and obtaining pelvis radiographs for adolescents with hip, thigh, or knee pain and a limp may reduce progression to more severe deformity.

This study has limitations. The retrospective design relies on clinical documentation for symptom onset and duration and may be subject to misclassification. Additionally, this is a single-center study that received referrals from a wide area outside of the immediate surroundings. This may introduce bias that limits external generalizability. However, this tertiary-care referral model is extremely prevalent across the United States with larger academic centers taking many SCFE cases when surrounding hospitals do not have sufficient pediatric orthopedic coverage. Symptom duration may be influenced by recall bias and differential access to care. ADI is a neighborhood-level measure and may not reflect individual household socioeconomic status. The cohort reflects cases captured through participating systems and may not represent all SCFE cases statewide. Finally, the observational design does not permit causal inference regarding the relationship between deprivation and severity.

## 5. Conclusions

SCFE presentation volume in this single-center cohort demonstrated significant seasonality, with a higher volume from midsummer through midwinter. Mean ADI and slip severity also varied seasonally, with lower-volume months characterized by greater neighborhood deprivation and more severe slips. ADI showed mild associations with BMI and slip severity, whereas BMI did not predict slip severity. Together, these findings support year-round vigilance for SCFE and underscore the importance of access to timely evaluation and imaging.

## Figures and Tables

**Table 1:** Comparisons of demographic and clinical characteristics among four seasons (median (IQR), *p* value).

Characteristic	Overall, N = 122 ^1^	Fall, N = 38 ^1^	Spring, N = 18 ^1^	Summer, N = 35 ^1^	Winter, N = 31 ^1^	*p*-Value ^2^
ADI rank ^1^	68 (43, 88)	58 (37, 87)	76 (51, 85)	74 (53, 91)	57 (35, 79)	0.3
Height ^1^	0.89 (0.71, 0.97)	0.90 (0.70, 0.97)	0.72 (0.48, 0.91)	0.90 (0.77, 0.96)	0.89 (0.71, 0.98)	0.08
Weight ^1^	0.99 (0.95, 1.00)	0.98 (0.94, 1.00)	0.99 (0.96, 0.99)	0.99 (0.98, 1.00)	0.99 (0.95, 1.00)	0.4
BMI score ^1^	0.98 (0.95, 1.00)	0.96 (0.94, 1.00)	0.97 (0.97, 0.98)	0.99 (0.97, 1.00)	0.98 (0.95, 0.99)	0.5
Side(s) affected						0.8
Bilateral	31 (25%)	7 (18%)	4 (22%)	11 (31%)	9 (29%)	
Left	54 (44%)	18 (47%)	8 (44%)	13 (37%)	15 (48%)	
Right	37 (30%)	13 (34%)	6 (33%)	11 (31%)	7 (23%)	
Temporal classification						0.2
Acute	28 (23%)	8 (21%)	1 (5.6%)	7 (20%)	12 (41%)	
Acute on chronic	25 (21%)	7 (18%)	4 (22%)	8 (23%)	6 (21%)	
Chronic	67 (56%)	23 (61%)	13 (72%)	20 (57%)	11 (38%)	
Loder class						0.4
Stable	84 (69%)	28 (76%)	14 (78%)	21 (60%)	21 (68%)	
Unstable	37 (31%)	9 (24%)	4 (22%)	14 (40%)	10 (32%)	
Vit D prior to diagnosis ^1^	26 (20, 29)	29 (25, 34)	21 (21, 21)	20 (20, 27)	26 (23, 26)	0.4
Duration of symptoms ^1^	30 (14, 90)	60 (21, 90)	75 (18, 304)	29 (16, 60)	14 (7, 75)	0.018
Triradiate cartilage						>0.9
Closed	36 (30%)	12 (32%)	5 (28%)	9 (26%)	10 (32%)	
Open	86 (70%)	26 (68%)	13 (72%)	26 (74%)	21 (68%)	

^1^ Median (IQR); n (%). ^2^ Kruskal–Wallis rank sum test; Fisher’s exact test; Pearson’s chi-squared test.

**Table 2:** Relationship between ADI and SSA or BMI using linear regression analysis (r, *p* value).

Predictor	Outcome	r	*p*-Value	N
ADI	Southwick Slip Angle	0.279	0.013	77
ADI	BMI	0.315	0.002	94
BMI	Southwick Slip Angle	0.114	0.285	89

## Data Availability

De-identified data are available from the corresponding author upon reasonable request and with appropriate institutional approvals.

## Abbreviations

The following abbreviations are used in this manuscript:

SCFE	Slipped Capital Femoral Epiphysis
ADI	Area Deprivation Index
BMI	Body Mass Index
SSA	Southwick Slip Angle
